# The Application of Sonovaginography for Implementing Ultrasound Assessment of Endometriosis and Other Gynaecological Diseases

**DOI:** 10.3390/diagnostics12040820

**Published:** 2022-03-27

**Authors:** Francesca Arezzo, Gennaro Cormio, Daniele La Forgia, Adam Abdulwakil Kawosha, Michele Mongelli, Carmela Putino, Erica Silvestris, Donato Oreste, Claudio Lombardi, Gerardo Cazzato, Ettore Cicinelli, Vera Loizzi

**Affiliations:** 1 Obstetrics and Gynecology Unit, Department of Biomedical Sciences and Human Oncology, University of Bari “Aldo Moro”, Piazza Giulio Cesare 11, 70124 Bari, Italy; michelemongelli1992@gmail.com (M.M.); carmen.putino@hotmail.it (C.P.); dr.claudiolombardi@gmail.com (C.L.); ettore.cicinelli@uniba.it (E.C.); 2 Obstetrics and Gynecology Unit, Interdisciplinar Department of Medicine, University of Bari “Aldo Moro”, Piazza Giulio Cesare 11, 70124 Bari, Italy; gennaro.cormio@uniba.it (G.C.); vera.loizzi@uniba.it (V.L.); 3 SSD Radiodiagnostica Senologica, IRCCS Istituto Tumori “Giovanni Paolo II”, Via Orazio Flacco 65, 70124 Bari, Italy; d.laforgia@oncologico.bari.it; 4 Department of General Medicine, Universitatea Medicina si Farmacie Grigore T Popa, Strada Universitatii 16, 700115 Iasi, Romania; adam.akawosha@gmail.com; 5 Gynecologic Oncology Unit, IRCCS Istituto Tumori “Giovanni Paolo II”, Via Orazio Flacco 65, 70124 Bari, Italy; ericasilvestris@gmail.com; 6 SSD Radiologia Diagnostica, IRCCS Istituto Tumori “Giovanni Paolo II”, Via Orazio Flacco 65, 70124 Bari, Italy; d.oreste@oncologico.bari.it; 7 Pathology Section, Department of Emergency and Organ Transplantation, University of Bari “Aldo Moro”, Piazza Giulio Cesare 11, 70124 Bari, Italy; gerycazzato@hotmail.it

**Keywords:** sonovaginography, endometriosis, gynaecological ultrasound, vaginal diseases, cervical diseases

## Abstract

Sonovaginography is a way of assessing gynaecological diseases that can be described as cheap yet accurate and non-invasive. It consists of distention of the vagina with ultrasound gel or saline solution while performing transvaginal sonography to clearly visualize and assess a host of local cervical, as well as any vaginal, disorders. With endometriosis being a steadily growing gynaecological pathology affecting 8–15% of women of fertile age, transvaginal sonography (TVS) can be considered as one of the most accurate and comprehensive imaging techniques in its diagnosis. Nevertheless, the accuracy may vary depending on scan sites. The purpose of this narrative review is to assess the performance of sonovaginography in detecting endometriosis in those sites where TVS has a low sensitivity.

## 1. Introduction

There are several sonographic as well as anatomic factors that may impact the visualisation of the vagina and the distal cervix. When performing a sonography, the endovaginal transducer goes into the vaginal fornices, which makes it difficult to properly examine the vagina given the similar echogenicities between cervical and vaginal pathology. Anatomic factors include the uterine position, which determines the orientation of the cervix relative to the vagina; the degree of bladder distention; and the pelvic floor strength. The variable orientation of the cervix relative to the vagina is dependent upon uterine position, the degree of bladder distention, and pelvic floor strength. These factors create critical angle artifacts, obscuring the junction of the cervix and vagina [1].

Sonovaginography (SVG) is a useful and accurate technique to visualise and assess a host of cervical and vaginal disorders that entails the distention of the vagina with ultrasound gel or saline solution while performing transvaginal sonography (TVS).

While speculum examination only allows one to visualise the surface of the lesions of abnormalities in the vicinity, sonography also shows the thickness, the extension, the vascularity, and the echo texture. In some people, during a speculum examination, it is difficult to visualise the cervix, as it is localised deep in the vaginal vault [2]. A speculum examination is also difficult to perform in postmenopausal women and in those with infiltrating lesions, since the vaginal walls are characteristically less elastic, which may cause pain. On the other hand, using a distension medium in the vagina induces passive distension of the vaginal walls (as opposed to forceful distension in a speculum examination) [3].

Endometriosis a steadily growing gynaecological pathology affecting 8–15% of women of fertile age. This disease is characterised by the development of endometrium-like tissue outside the uterine cavity, provoking pain during or outside the menstrual cycle [4].

TVS can be considered as one of the most accurate and comprehensive imaging techniques in the diagnosis of endometriosis [5,6]. The approach to sonographic evaluation in women who may be affected by endometriosis was established by the International Deep Endometriosis Analysis (IDEA) group.

The method is particularly efficient in diagnosing ovarian endometriomas and urinary bladder endometriosis, but its sensitivity is lower when it comes to endometriotic lesions in the posterior pelvic compartment, vagina, uterosacral ligaments, and rectovaginal septum [7,8].

The purpose of this narrative review is to analyse the performance of sonovaginography in the detection of endometriosis in those sites where TVS has a low sensitivity.

## 2. Methods

In December 2021, we searched MEDLINE and Scopus for randomised controlled trials; narrative and systematic reviews; meta-analyses; observational studies, either longitudinal or historical; and case series published in English in the last 25 years using keywords sonovaginography; endometriosis; gynaecological ultrasound; vaginal diseases; and cervical diseases. For this narrative review, abstracts from 96 manuscripts found in the literature were assessed by two independent authors; of these, 48 (9 sonovaginography, 12 endometriosis, 16 gynaecological ultrasound, 4 vaginal diseases, and 7 cervical diseases) were included, based on the impact of the latter studies on current patient management.

## 3. Procedures

The technique involves the use of an approximately 60 cc Luer-lock syringe that is filled with saline solution or water-based gel suitable for ultrasonography. The examiner parts the labia with his fingers and holds the syringe firmly against the introitus to prevent leakage while infusing the saline solution. When examining small, stenotic, or obstructed vagina canals, it is recommended to use a lower quantity of ultrasound gel (or even saline, to prevent a rapid reflux out of the vagina given the lower viscosity of saline). To distend a severely restricted vaginal canal, the examiner can use a smaller syringe, Foley catheter, or angiocath [1].

The distension medium partially distends the vagina, including the fornices, and allows the examiner to better visualize the inner vaginal contour as well as the relationships between the various structures, namely the vaginal walls and the cervix.

The ultrasound gel or saline solution generates an acoustic window, which reinforces the contrast to better assess not only the vaginal walls, vaginal fornices, and cervix, but also adjoining structures such as the pouch of Douglas, rectovaginal septum, anterior rectal wall, paravaginal and parametrial tissues, and the bladder [3]. The exam is normally tolerated by patients, with no mentionable complications.

## 4. Applications of Sonovaginography in the Assessment of Endometriosis

**Anterior pelvic compartment**

When investigating the anterior pelvic compartment, the urinary bladder wall (muscular and mucous) is investigated as well as the distal ureters, intramural part of the ureter, and pelvic part of the ureters (4–6 cm distance above the ureterovesical junction).

The detection of hypoechoic nodules with regular or irregular contours or linear hypoechoic thickenings in the walls of the bladder, or in the vesicouterine space, may indicate the presence of endometriotic lesions [7].

**Posterior pelvic compartment**

In the posterior pelvic compartment, it is possible to evaluate the posterior wall of the vagina, the posterior side of the uterus and cervix, the uterosacral ligaments, the rectovaginal septum, and anterior walls of the rectosigmoid. The transvaginal probe is normally inserted in the posterior vaginal fundus. The examiner places one hand on the lower abdominal wall to mobilise the uterus, and with the other hand, he applies a slight pressure on the transvaginal probe. In this way, the examiner can evaluate the mobility of the Douglas pouch, the presence of uterorectal adhesions, and the obliteration of the Douglas pouch. The examination of the posterior part of the uterus and the uterosacral ligaments should be done during the withdrawal of the probe from the posterior vaginal fundus.

The sonographic evaluations of the pelvic anatomical structures that may indicate the presence of endometriosis include hypoechoic linear thickenings, asymmetry of the uterosacral ligaments, and tumoural masses with regular or irregular contour. When it comes to investigating the posterior vagina, the rectosigmoid, and the rectovaginal septum, the examiner should move the probe cranially and caudally on the posterior vaginal wall while making rotating movements on different planes. The presence of endometriosis in the vagina can be identified by the existence of thickenings and/or cystic or non-cystic nodular lesions with hypoechoic aspect situated in the posterior vaginal wall [7].

The application of SVG in the detection of endometriosis was first described in 2003 by Dessole et al. The study focused on 46 women who were selected for laparatomic or laparoscopic surgery because of the suspicion of rectovaginal endometriosis based on their history and/or clinical examination.

All the women were evaluated with TVS and then SVG with saline solution before undergoing the surgery. The study showed that SVG was more accurate in diagnosing rectovaginal endometriosis than TVS, with a sensitivity and specificity of 90.6% and 85.7% for SVG and 43.7% and 50% for TVS [9] (Table 1).

Brătilă et al. reported a multicentre study involving 193 women with endometriosis-related symptoms, who were evaluated with TVS and SVG with gel and subjected to laparoscopic surgery afterwards. The study found that the laparoscopy results were comparable to the ultrasound findings [7].

The sensitivity of TVS and SVG with gel in diagnosing the endometriosis of the anterior pelvic compartment shows no significant statistical difference (80.1% and 81.2%, respectively). Based on the same results, SVG with gel is more efficient in detecting the lesions of the posterior pelvic compartment, with a sensitivity of 85.3% compared to 73.1% obtained by means of TVS (*p* = 0.051).

The study also proved that SVG and TVS are less efficient in the diagnosis of lesions of the urinary bladder (sensitivity of 65.5% for TVS and 67% for SVG). Such findings can be explained by the fact that some patients had a semi-full or empty bladder or that the lesions were too small to be detected.

Another multicentre prospective observational study by Reid et al. [10] included 189 women subjected to preoperative gel SVG and laparoscopy for endometriosis.

The study proves that office gel SVG seems to be successful in the prediction of deep-bowel-infiltrating endometriosis (DIE), with a higher accuracy (94.7%) in rectosigmoid compared with anterior rectal DIE (92%).

For posterior vaginal wall (PVW) and rectovaginal septum (RVS) DIE, the accuracy was 95% and 95%, respectively. The sliding sign had a sensitivity of 85% in the prediction of POD obliteration.

Therefore, a negative gel SVG examination indicates the absence of DIE at laparoscopy.

In a study by Saccardi et al. [11], the authors analysed 54 patients affected by posterior deep pelvic endometriosis, comparing the roles of TVS, SVG, and MRI. Their analysis reported that SVG and MRI provide a more reliable diagnosis by also defining the precise localisation of endometriotic lesions, with respective sensitivities of 94.7 and 73.1% for vaginal fornix, 88.9 and 66.7% for the uterosacral ligaments, and 80.6 and 83.3% for involvement of the rectovaginal septum. SVG and MRI showed a specificity of 97.1 and 94.3% for vaginal fornix, 95.6 and 95.6% for uterosacral ligaments, and 100 and 77.8% for involvement of the rectovaginal septum, respectively. SVG and MRI also showed a specificity of 93.8% and 95.8%, respectively, as well as a sensitivity of 66.7% for both in the diagnosis of rectal endometriosis

Hence, they concluded that TVS should preferably be used as a diagnostic technique of posterior DIE whilst SVG and/or MRI should be used for further investigations.

Exacoustos et al. [12] analysed 50 patients with deep pelvic endometriosis who were evaluated using TVS alone or SVG associated with transrectal sonography (TRS) before undergoing a laparoscopic surgery. The sensitivity in the use of TVS alone (88, 90%) was similar to the association of TRS and SVG (89, 90%) in the diagnosis of endometriotic nodules of the uterosacral ligament (USL) and of the distal rectal and sigmoid wall. However, the use of TRS associated with SVG shows a much higher accuracy in the diagnosis of rectovaginal nodules, especially when they infiltrate the RVS and posterior vaginal fornix, compared to TVS alone (TVS alone 67% vs. 91%).

Hence, TVS/TRS and sonovaginography could be as accurate as MRI in providing accurate information on the presence and the extension of pelvic DIE when performed by a well-trained sonographer.

Reid et al. conducted a study using intra-operative saline SVG to define the rectovaginal septum in 23 women with suspected rectovaginal endometriosis. The study took into consideration the thickness of the RVS at three different anatomical sites: the posterior fornix (retrocervical area), the middle third of the vagina (upper RVS), and just above the perineal body (lower RVS). The thickness of the RVS was not significantly different in a woman with endometriosis [13].

Although the sample of women studied is small, the study proved that there is no correlation between RVS thickness and the presence of rectovaginal nodules.

Finally, Barra et al. [14], in 2021, investigated 281 patients with suspicion of posterior DIE by performing rectal water-contrast transvaginal ultrasonography (RWC-TVS) and SVG.

Performing the RWC-TVS, they injected 300 mL circa of saline solution by using a catheter connected to a 100 mL sterile syringe in the rectum, up to a distance of approximately 15 cm from the anal verge, to distend the rectosigmoid under ultrasonographic control.

RWC-TVS and SVG had similar sensitivity (93.8% vs. 89.4%; *p* = 0.210) in diagnosing DIE of the posterior pelvic compartment, and both techniques showed good comparable diagnostic accuracy (91.8% vs. 86.8%; *p* = 0.775). Both exams can correctly detect implants in uterosacral ligaments, the vagina, and the rectovaginal septum. However, RWC-TVS proved to be better in detecting the presence and describing the characteristics of the rectosigmoid endometriosis (with a sensitivity of 95.2% vs. 82.0%; *p* = 0.003) by better estimating the intestinal submucosa infiltration, as well as the distance between the nodule and anal verge.

### 4.1. Other Applications

#### 4.1.1. Applications of Sonovaginography in the Assessment of Vaginal Diseases

At ultrasound examination, the vagina can be seen, in the midline sagittal plane, as a collapsed, hypoechoic tubular structure with a central hyperechoic echo, representing the apposed surfaces of the vaginal mucosa. In menopause, the vagina becomes shorter and the mucosa gets less echogenic due to diminished oestrogen stimulation [15].

**Vaginal cysts**

**Figure 1:**
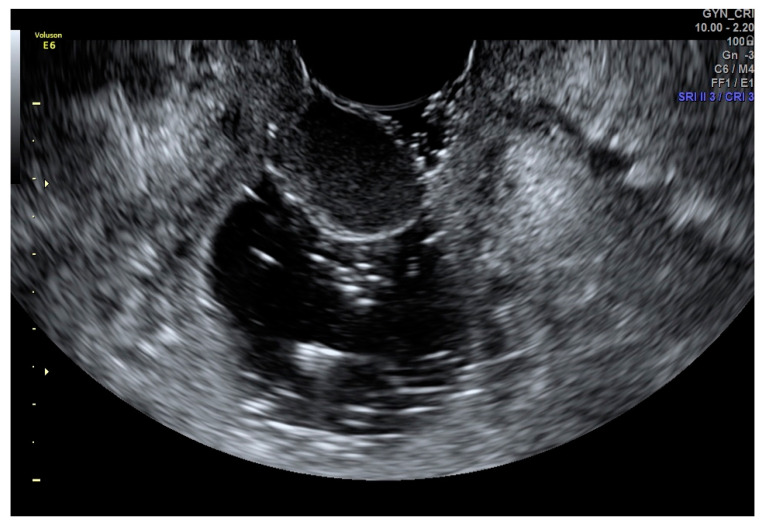
SVG image of a vaginal cyst.

The ultrasound of vaginal cysts shows an anechoic or echogenic thin-walled cyst. Cysts within the proximal vaginal wall are likely to resemble the cervix. Anterior wall vaginal cysts could also be confused with urethral diverticula. However, with sonovaginography, there is a clear demonstration of the origin of these cysts from the vaginal wall rather than being seen as parts of the cervix or urethra (Figure 1) [16].

**Vaginal Polyps**

Using conventional sonographic techniques, these masses are inconspicuous and ill-defined, whereas an isoechoic polypoid mass that appears to be jutting directly out of the vaginal wall can be detected using sonovaginography, with the color Doppler to define a central vessel into the polypid mass [17].

**Vaginal Leiomyoma**

**Figure 2:**
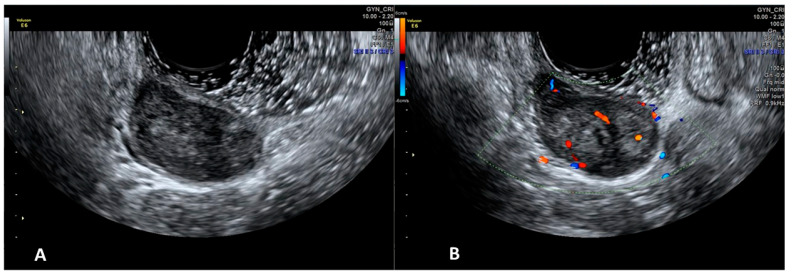
SVG image of a vaginal leiomyoma (Panel (**A**)) an its vascularization (Panel (**B**)).

The sonographic appearance of vaginal leiomyomas could easily be mistaken as uterine fibroids. They are usually intramurally situated hypoechoic masses with regular walls and posterior acoustic shadowing and calcifications [16,17]. With sonovaginography, the mass can be more clearly seen within the muscular layer of the vaginal wall (Figure 2). This is essential to being able to differentiate a vaginal wall leiomyoma from the more common prolapse of a submucosal uterine leiomyoma into the vaginal canal [17].

**Vaginal Stenosis**

Sonovaginography shows the length, the level, and the severity of the tightening of the vaginal canal. In case of vaginal stenosis, saline is preferred to ultrasound gel to prevent chronic retention of inspissated gel [17].

**Condylomata Acuminata**

The sonovaginography evidences the condyloma as an irregular stalk that grows on the vaginal wall with increased vascularity on Doppler [17].

**Vaginal Cancer**

The sonovaginography highlights a thickened irregular vaginal wall or infiltrative mass that appears to jutting directly out of the vaginal wall into the vaginal lumen. A vaginal wall mass presenting scarcely defined margins but a smoothly marginated surface is more likely to be metastatic. Chronic inflammatory changes caused by previous surgery, infection, or radiation might also resemble malignancy [17].

#### 4.1.2. Applications of Sonovaginography in the Assessment of Urethral Diseases

On ultrasonography, the urethra looks like a tubular structure with a central echolucent urethral mucosa and adjoining hyperechogenic urethral sphincters [18].

**Urethral Diverticulum**

In transvaginal or transperineal ultrasound, a periurethral lesion with cystic appearance would be observed in continuity with the urethra. The sonovaginography better defines the anatomic relationship between the urethra, the cystic mass, and the vaginal wall [1].

#### 4.1.3. Applications of Sonovaginography in the Assessment of Cervical Diseases

The cervix has a similar echogenicity to the myometrial tissue of the uterine body with a homogeneous echotexture. The cervical mucosa is usually hypoechoic with the endocervical glands. In the nongravid state, the uterine cervix measures approximately 25 mm in length and 20–25 mm in total width [19,20].

**Cervical Polyps**

Cervical polyps within the endocervical canal may be recognized during a conventional transvaginal sonography. The sonovaginography is the best technique to describe a polyp or polyps coming out from the cervical os and penetrating into the vaginal canal. The sonovaginogram is also indicated in the case of suspicion of a mass at the external os on either physical exam or transvaginal sonography. The possibility of a polyp can be increased by the presence of an abnormal vessel seen on colour Doppler imaging within the cervical canal. Cervical polyps look like an ovoid or finger-like hypoechoic or hyperechoic mass with cystic characteristics coming out from the external cervical os. A central feeding artery and draining vein are visible in the polyp on colour Doppler imaging. A dynamic palpation with an endovaginal transducer typically demonstrates a soft, mobile, deformable mass [17].

**Cervical Leiomyoma**

Sonography, and especially sonovaginography, can define the size, the degree of prolapse, and the site of origin of the pedicle of the leiomyoma better than a physical exam. These factors facilitate the preoperative surgical planning, such as hysteroscopic versus direct vaginal myomectomy, and help assess the necessity for cervical dilatation [21,22].

**Cervical Cancer**

The role of ultrasound in the evaluation of carcinoma has been proven efficient by several studies. Due to the fact that the vaginal walls are collapsed, their similar echogenicities, which reduce the contrast resolution, limit the discrimination between cervical cancer and the vagina, making the ultrasound evaluation of exophytic early-stage cervical cancer often difficult [23,24,25].

**Figure 3:**
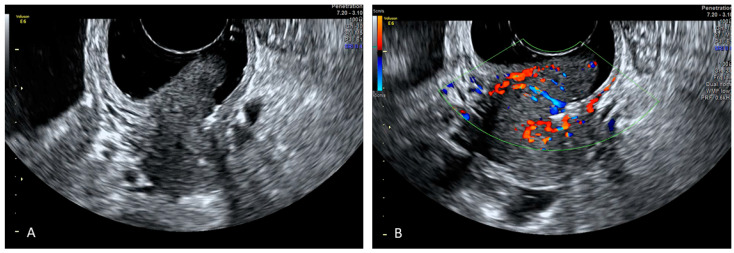
SVG image of an exophytic cervical cancer lesion (Panel (**A**)) and its vascularization (Panel (**B**)).

**Figure 4:**
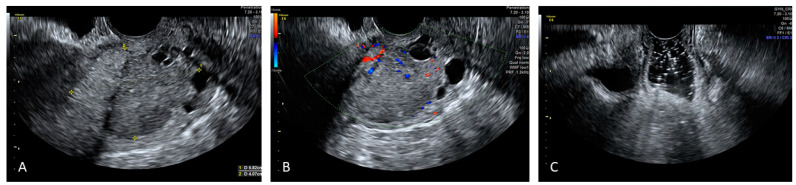
Reports the SVG image of a mixed echogenicity cervical cancer lesion whose caudal limit is delimited by nabothian cysts (Panel (**A**)) and its vascularization (Panel (**B**)). SVG reveals regular vaginal walls (Panel (**C**)).

Although there are no studies in the literature to date, SVG may be a simple and cheap procedure for the evaluation of patients with mostly exophytic early cervical cancers when TVS alone is not adequate for the correct assessment of the tumour, its size, and its fornices infiltration (Figure 3 and Figure 4).

#### 4.1.4. Applications of Sonovaginography in the Assessment of Mullerian Anomalies

**Septate vagina**

The assessment is achieved using gel sonovaginography, with the gel instilled in each of the hemivaginas. The SVG confirms the presence of the septum, the positions of the two hemivaginas relative to each other, the thickness and length of the septum, and the location of the external cervical os [3].

**Duplicate cervix**

With sonovaginography and the presence of the distension medium in the vagina, it may be easier to visualize the profile of a double cervix.

## 5. Conclusions

SVG is a simple, well-tolerated, and inexpensive technique that, if performed by experienced sonographers, can improve the evaluation of different gynecological diseases. It is also a dynamic test that can be executed directly by the gynecologist.

Looking at the distention mediums in SVG, the use of gel could be preferable, as it only requires one examiner, whilst saline needs a second examiner to close the labia and avoid possible leakage outside the vagina, which may result in minimum discomfort of the patient [10].

However, nowadays, literature data have evaluated the performance of this technique only in the evaluation of endometriosis. We hope that studies evaluating the application of this simple method in other gynaecological diseases will be carried out soon.

## Figures and Tables

**Table 1:** Performance of sonovaginography in the detection of endometriosis.

Author	Year	Population	Distension Medium	Sites of Endometriosis	TVS Sensitivity	SVG Sensitivity	RMI Sensitivity	RWC-TVS Sensitivity	TVS Specificity	SVG Specificity	RMI Specificity	TVS Accuracy	SVG Accuracy	RWC-TVS Accuracy
Dessole et al. [9]	2003	46	saline solution	RVS	43.7%	90.6%			50%	85.7%				
Brătilă et al. [7]	2016	193	gel	anterior pelvic compartment	80.1%	81.2%								
				posterior pelvic compartment	73.1%	85.3%								
				urinary bladder	65.5%	67%								
Reid et al. [10]	2014	189	gel	rectosigmoid		84.6%							94.7%	
				anterior rectal DIE		72.2%							92%	
				PVW		18.2%							95%	
				RVS		18.2%							95%	
Saccardi et al. [11]	2012	54	saline solution	vaginal fornix		94.7%	73.1%			97.1%	94.3%			
				USL		88.9%	66.7%			95.6%	95.6%			
				RSV		80.6%	83.3%			100%	77.8%			
				rectal endometriosis		66.7%	66.7%			93.8%	95.8%			
Exacoustos et al. [12]	2008	50	gel	USL	88%,	90%								
				distal rectal										
				sigmoid wall										
				RSV								67%	91%	
				PVW										
Barra et al. [13]	2021	281	gel	posterior pelvic compartment		89.4%		93.8%					86.8%	91.8%

TVS: transvaginal sonography; SVG: sonovaginography; RMI: magnetic resonance imaging; RWC-TVS: water-contrast transvaginal ultrasonography; DIE: deep-bowel-infiltrating endometriosis; USL: uterosacral ligaments; PVW: posterior vaginal wall; RVS: rectovaginal septum; USL: uterosacral ligament.

## Data Availability

Not applicable.
